# Vitamin D alleviates type 2 diabetes by promoting autophagy and inhibiting inflammation via the NLRP3 inflammasome pathway

**DOI:** 10.1515/biol-2025-1294

**Published:** 2026-03-05

**Authors:** Qian Ren, Ling Zhang, Chengpei Ni, Lewen Zhang, Yudie Hu, Min Xiao, Zhengyu Zhou

**Affiliations:** Laboratory Animal Center of Suzhou Medical College, Soochow University, Suzhou 215123, China; Suzhou Kezhuo Medical Technology Co., Ltd, Suzhou 215000, China; Wuxi Center for Disease Control and Prevention, Wuxi 214023, China; CCIC Huatongwei International Inspection Co., Ltd, Suzhou 215123, China; Second Affiliated Hospital of Soochow University, Suzhou 215004, China

**Keywords:** vitamin D, autophagy, inflammatory response, type 2 diabetes mellitus, molecular mechanism

## Abstract

We investigated whether vitamin D (VD) alleviates type 2 diabetes mellitus (T2DM) by modulating autophagy and inflammation. In wild-type diabetic mice, VD supplementation significantly improved glucose tolerance, reduced fasting blood glucose, and the HOMA-IR (homeostasis model assessment of insulin resistance) index (P < 0.05). Serum levels of IL-1β, TNF-α, and tissue reactive oxygen species were markedly elevated in T2DM mice but significantly decreased after VD treatment (P < 0.05). Histopathological and ultrastructural analyses revealed that VD preserved pancreatic and kidney tissue integrity and increased autophagic structures. Consistently, VD upregulated Beclin-1 and LC3-II while downregulating IL-1β and NF-κB p65 expression in these tissues (P < 0.05). In contrast, these beneficial effects of VD were largely absent in NLRP3-knockout T2DM mice. Collectively, vitamin D exerts therapeutic effects in T2DM by promoting autophagy and inhibiting inflammation, primarily through the ROS-NLRP3-IL-1β-NF-κB signaling pathway.

## 1 Introduction

Diabetes mellitus (DM) represents a significant global public health concern, with a dramatically increasing prevalence, morbidity, and mortality [1], 2]. According to the International Diabetes Federation, 463 million individuals were diagnosed with DM in 2019, and this number is estimated to increase to 783.2 million worldwide in 2045 [3]. Notably, in mainland China, a 2017 national epidemiological survey reported a DM prevalence of 12.8 % by using the American Diabetes Association diagnostic criteria [4]. Type 2 diabetes mellitus (T2DM), accounting for the majority of all DM cases, is characterized by elevated fasting blood glucose (FBG) levels resulting from impaired insulin secretion, insulin resistance (IR) [5], 6], and β-cell dysfunction [7]. T2DM is associated with many chronic complications, including cardiovascular and chronic kidney diseases, leading to higher morbidity, mortality, and huge economic burdens on healthcare systems [8]. The inflammation hypothesis proposes that chronic, low-level inflammation is a key contributor to the pathogenesis of DM, quantifiable through the analysis of inflammatory markers [9], 10].

Vitamin D (VD) deficiency is more common in patients with diabetes than in those without [11]. VD deficiency can impair autophagic flux, a process essential for clearing damaged organelles and proteins [12] and, importantly, involved in T2DM pathophysiology [13], [14], [15]. This impairment contributes to β-cell dysfunction and IR [16]. Previous studies have demonstrated that VD can reduce inflammation in the pancreas and kidneys, enhance autophagy, and alleviate T2DM symptoms [17], 18]. Furthermore, recent clinical evidence and reviews strongly support the role of VD supplementation in improving glycaemic control and its complex interplay with inflammatory pathways in T2DM [19], 20]. These findings lead us to hypothesize that VD may participate in the alleviation of diabetes by modulating pathways associated with both autophagy and inflammation.

NLRP3 plays a critical role in chronic low-grade inflammation and is a key component of the innate immune system. The NLRP3 inflammasome senses metabolic danger signals, such as hyperglycemia and free fatty acids, thereby activating caspase-1 and promoting the maturation and secretion of IL-1β. As a potent pro-inflammatory cytokine, IL-1β not only directly interferes with insulin signaling pathways, leading to IR, but also amplifies inflammatory responses through the activation of the NF-κB signaling pathway, creating a vicious cycle [21], 22]. Furthermore, the inflammatory milieu in T2DM is characterized by the elevation of multiple cytokines, including the NLRP3-dependent IL-18, the NF-κB-driven TNF-α, and the T-cell-derived IFN-γ, all of which contribute to IR and β-cell dysfunction. A recent randomized clinical trial directly demonstrates that vitamin D3 supplementation reduces oxidative stress and downregulates NLRP3 gene expression in patients with T2DM, highlighting this pathway’s clinical relevance [23]. However, whether NLRP3 is a necessary mediator for the therapeutic role of VD in T2DM remains to be fully determined.

Herein, we aimed to investigate the molecular mechanisms by which VD modulates autophagy and the inflammatory response in T2DM, with a specific focus on the essential role of the NLRP3 inflammasome. Using wild-type (WT) and NLRP3-knockout (Nlrp3^−/−^) mice, we examined key metabolic tissues (pancreas, kidney, and liver) and systemic inflammation. Our findings identify NLRP3 as a crucial target of VD, provide a mechanistic link between its antioxidant, pro-autophagic, and anti-inflammatory actions, and support VD supplementation as a potential strategy for T2DM.

## 2 Methods

### 2.1 Study animals

WT C57BL/6 male mice (*n* = 30; 3 weeks old) were obtained from Shanghai SLAC Laboratory Animal Co., Ltd (China). C57BL/6J male mice with Nlrp3^−/−^ (*n* = 30; 3 weeks old) were gifted by the Cambridge-Su Genome Resource Center (Suzhou, China). The mice were housed in specific pathogen-free conditions at Soochow University (Suzhou, China), maintained on a 12-h light/dark cycle, at a temperature of 22 ± 2 °C, and a humidity of 55 ± 5 %. Animals were allowed free access to water and standard rodent feed.

**Ethical approval:** The research related to animal use has been complied with all the relevant national regulations and institutional policies for the care and use of animals, and has been approved by the Animal Ethics Committee of Laboratory Animals of Soochow University (approval No. 202206A0674).

### 2.2 Induction of T2DM and experimental design

T2DM was induced using a high-fat diet (HFD) combined with low-dose streptozotocin (STZ) (Sigma-Aldrich, St. Louis, MO, USA; Cat. No. S0130) injections, following an established protocol with minor modifications [24]. In detail, WT and Nlrp3^−/−^ mice were subjected to an HFD (Suzhou Shuangshi Experimental Animal Feed Co., Ltd, China), which consisted of 50 % standard diet, 5 % soybean powder, 5 % casein, 10 % milk powder, 5 % peanuts, 5 % egg yolk powder, 12 % lard, 5 % sucrose, 1 % sesame oil, 2 % salt, and 10 drops of vitamin A. The fraction of each component represents its percentage of the total weight. The control mice received a standard diet. Following 5 weeks on the HFD, mice were intraperitoneally injected with 50 mg/kg STZ dissolved in citrate buffer, once daily for 4 days [25]. Control mice were given equivalent amounts of citrate buffer and normal saline concurrently. Seven days after the last STZ injection, blood glucose concentrations were assessed following 12 h of fasting. Mice were classified as diabetic if their fasting plasma glucose levels were more than 11.1 mmol/L [26].

### 2.3 Animal treatment and grouping

WT and Nlrp3^−/−^ mice were divided into the WT group, WT-DM group, and WT-DM-VD group, as well as the Nlrp3^−/−^ group, Nlrp3^−/−^-DM group, and Nlrp3^−/−^-DM-VD group, respectively, with 10 mice in each group. Mice in the WT group and the Nlrp3^−/−^ group received a normal diet and intraperitoneal injection of citrate buffer and normal saline. The WT-DM group and the Nlrp3^−/−^-DM group were given an HFD diet and an intraperitoneal injection of STZ. Moreover, mice in the WT-DM-VD group and the Nlrp3^−/−^-DM-VD group were administered an HFD diet and intraperitoneal injection of STZ and VD (5 μg/kg; Sigma). The VD was given every other day for 3 weeks. The body weight, food and water intake, and blood glucose levels were monitored weekly for 21 days. Mice were sacrificed by cervical dislocation on day 22 after the last administration of VD.

### 2.4 Oral glucose tolerance test

On days 7, 14, and 21 after VD intervention, the mice in each group were fasted for 12 h but were allowed free access to water. Then, glucose (500 mg/mL; National Pharmaceutical Co., Ltd, Beijing, China) was administered by oral gavage (2 g/kg), and blood samples were collected via the tail vein at 0, 0.5, 1, and 2 h after glucose administration. FBG levels were measured using a Roche Vitality blood glucose detector (Roche Diagnostics GmbH, Mannheim, Germany). The area under the curve for glucose tolerance was calculated as follows: 1/4 (FBG at 0 h) + 1/2 (FBG at 0.5 h) + 3/4 (FBG at 1 h) + 1/2 (FBG at 2 h).

### 2.5 Analysis of the HOMA-IR index

On day 22 after VD intervention, blood samples were collected from the tail vein after 12 h of fasting. The serum was obtained after centrifugation. FBG levels were measured as described above. Additionally, fasting insulin levels were measured using a commercial Enzyme-Linked Immunosorbent Assay (ELISA) kit (Nanjing Jiancheng Bioengineering Institute, Nanjing, China; Cat. No. H203-1-1). The homeostasis model assessment of insulin resistance (HOMA-IR) index, a surrogate marker of systemic IR, was calculated as follows: HOMA-IR = (FBG (mmol/L) × fasting insulin (mIU/L))/22.5.

### 2.6 ELISA measurement of cytokines

On day 22 after the VD intervention, blood samples were collected from each group, and serum was isolated after centrifugation. The levels of inflammatory factors, including TNF-α, IL-18, IL-1β, and INF-γ, in the serum were measured using ELISA kits (Nanjing Jiancheng Bioengineering Institute, Nanjing, China; Cat. No. H052-1-1, H015-1-2, H002-1-2, and H025-1-2). We selected these specific cytokines based on their established roles in T2DM pathogenesis: IL-1β as the primary product of NLRP3 inflammasome activation; IL-18 as another key inflammasome-dependent cytokine; TNF-α as a major mediator of IR through NF-κB signaling; and IFN-γ as a representative T-helper 1 cytokine involved in systemic inflammation and β-cell dysfunction.

### 2.7 H&E staining

The pancreas and kidneys were dissected from mice of each group on day 22 after the last administration of VD. They were fixed in 10 % formalin for 2 h and then embedded in paraffin. Sections of 5 µm thickness were cut using a microtome, deparaffinized in xylene, and rehydrated through a graded ethanol series. Then, hematoxylin and eosin (H&E) staining was performed according to standard procedures. The stained sections were observed under the Leica DM300B microscope (Leica Microsystems, Germany). Qualitative histopathological evaluation of H&E-stained pancreatic and kidney sections was performed by two experienced pathologists who were blinded to the experimental groups. Assessments were based on consensus criteria for key features: islet architecture (intact, distorted, or shrunken), inflammatory infiltration (absent, mild, or severe), and glomerular morphology (normal, hypertrophic, or hypercellular).

### 2.8 Measurement of reactive oxygen species (ROS)

ROS levels in tissues were measured using a ROS Assay Kit (Beyotime Biotechnology, Shanghai, China; Cat. No. S0033S) with the 2′,7′-dichlorofluorescein-diacetate (DCFH-DA) probe [27]. After euthanizing the animals, the pancreas, kidneys, spleens, and livers were dissected and rinsed with cold PBS to remove blood and debris. The tissues were then homogenized. Tissue homogenates were centrifuged, and the resulting supernatant, containing intracellular components, was incubated with DCFH-DA in the dark at 37 °C. After 20 min, fluorescence intensity was measured at an excitation wavelength of 488 nm and an emission wavelength of 525 nm using a fluorescence spectrophotometer (BioTek, Winooski, VT, USA).

### 2.9 Western blotting

Proteins were extracted from mouse pancreatic tissues, separated using SDS-PAGE, and transferred to PVDF membranes. After blocking with 5 % non-fat milk for 1 h at room temperature, the membranes were probed overnight at 4 °C with the following primary antibodies: rabbit anti-mouse monoclonal Beclin 1 (Cell Signaling Technology, Danvers, MA, USA, Cat. No. 3495), rabbit anti-mouse monoclonal LC3-I/II (microtubule-associated protein 1A/1B-light chain 3) (Abcam, USA, Cat. No. ab192890), rabbit anti-mouse monoclonal IL-1β (Abcam, USA, Cat. No. Ab283818), rabbit anti-mouse monoclonal NF-κB p65 (Abcam, USA, Cat. No. Ab32536), and rabbit anti-GAPDH (Cell Signaling Technology, Cat. No. 5174). Subsequently, the membranes were incubated with horseradish peroxidase-conjugated goat anti-rabbit IgG (Beyotime Biotechnology, Cat. No. A0208) at room temperature for 90 min. Protein bands were visualized using the Enhanced Chemiluminescence Plus Western Blotting Detection system (GE Healthcare Bio-Sciences, Pittsburgh, PA, USA). The expression levels of LC3-I/II, Beclin-1, NF-κB p65, and IL-1β were normalized to GAPDH.

### 2.10 Electron microscopy

Electron microscopy analysis was performed as previously described [28], 29]. Briefly, the pancreas and kidney tissue samples were cut into small pieces, fixed in 2.5 % glutaraldehyde for 4 h, washed twice with 1 M phosphate buffer, and then fixed in 1 % OsO4 for 60 min. After dehydration in a graded acetone series, the samples were embedded for at least 2 h and made into ultrathin sections. These sections were then stained with lead nitrate and examined using an electron microscope (Talos F200S TEM, ThermoFisher). Ultrastructural features (e.g., secretory granule density, mitochondrial integrity, presence of double-membrane vesicles, podocyte foot process effacement) were evaluated by two independent researchers blinded to the groups, using standardized criteria and representative micrographs from multiple fields per sample.

### 2.11 Statistical analysis

Statistical analyses were conducted using SPSS version 17.0 (SPSS, Inc., Chicago, IL, USA). Data were obtained from a minimum of three independent experiments and are presented as mean ± standard deviation. The normality of data distribution was confirmed using the Shapiro-Wilk test. Group comparisons were performed using a chi-squared test or one-way analysis of variance followed by Tukey’s post hoc test. A significance level of P < 0.05 was considered indicative of statistically significant differences.

## 3 Results

### 3.1 Effects of VD on glucose metabolism and IR

**Figure 1:**
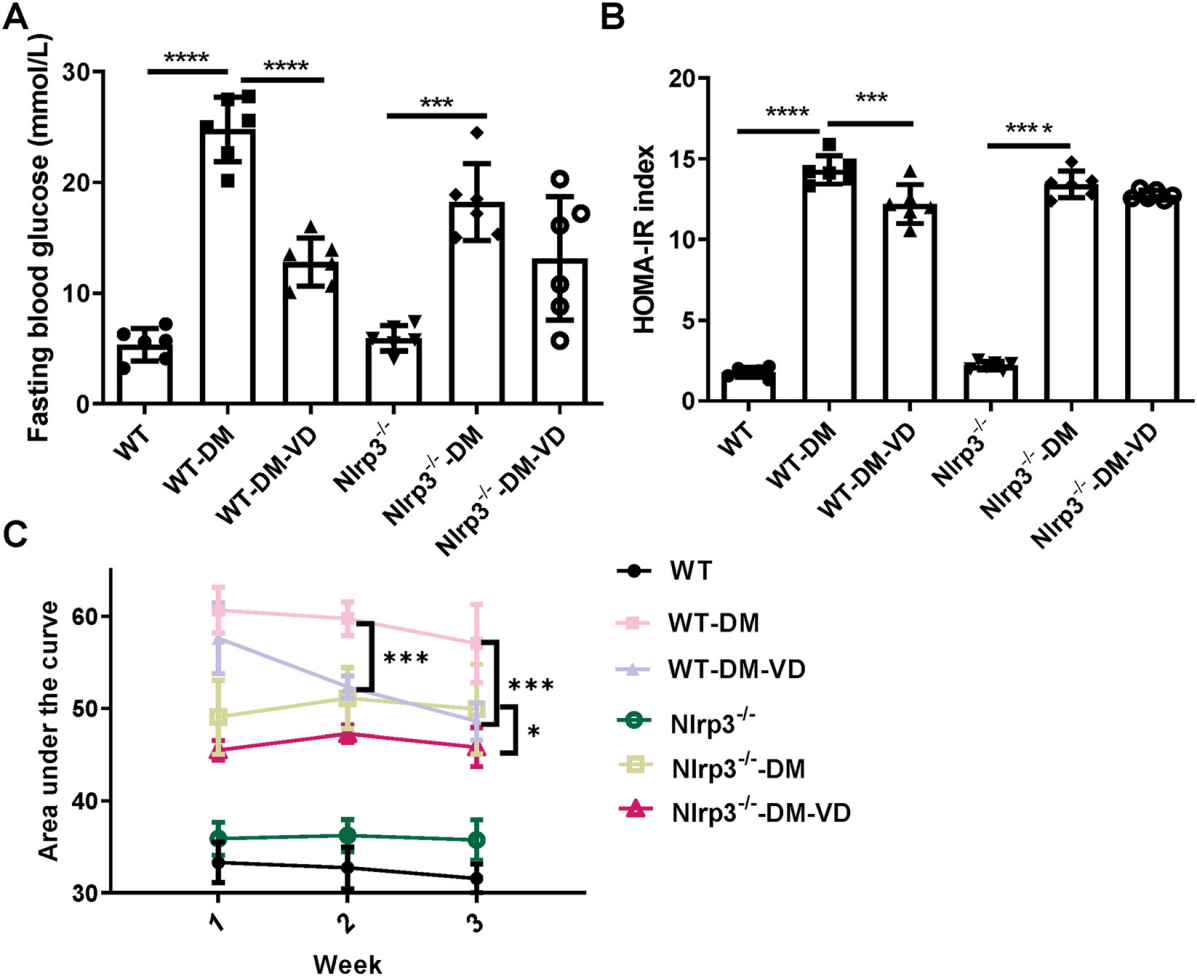
Analysis of FBG and the HOMA-IR index in different groups. (A) Comparison of FBG levels. (B) Comparison of the HOMA-IR index. (C) The area under the curve for the oral glucose tolerance test, measured at weeks 1, 2, and 3 after the initiation of VD intervention. Note: *P < 0.05, ***P < 0.001, ****P < 0.0001.

FBG and the HOMA-IR index were significantly elevated in both WT-DM and Nlrp3^−/−^-DM mice compared to their respective non-diabetic controls (P < 0.05; Figure 1A and B). VD treatment significantly attenuated these increases in WT-DM-VD mice (FBG and HOMA-IR: P < 0.001 vs. WT-DM). Specifically, the HOMA-IR index in WT-DM mice (13.99 ± 0.77) was 8.7-fold higher than in WT controls (1.61 ± 0.29), and VD intervention reduced it to 12.21 ± 1.20. In contrast, VD treatment did not significantly lower FBG or the HOMA-IR index in Nlrp3^−/−^-DM-VD mice compared to the Nlrp3^−/−^-DM group (P > 0.05), where the HOMA-IR index (13.43 ± 0.80 vs. 12.75 ± 0.36) remained 6.2-fold higher than its control (2.20 ± 0.26). Oral glucose tolerance, assessed by the area under the curve, was significantly improved in the WT-DM-VD group at weeks 2 and 3 post-intervention (P < 0.05; Figure 1C), while only a minor improvement was observed in the Nlrp3^−/−^-DM-VD group at week 3. These results indicate that VD’s ability to improve glucose metabolism and IR is largely dependent on the NLRP3 pathway.

### 3.2 Effects of VD on the levels of inflammatory cytokines

**Figure 2:**
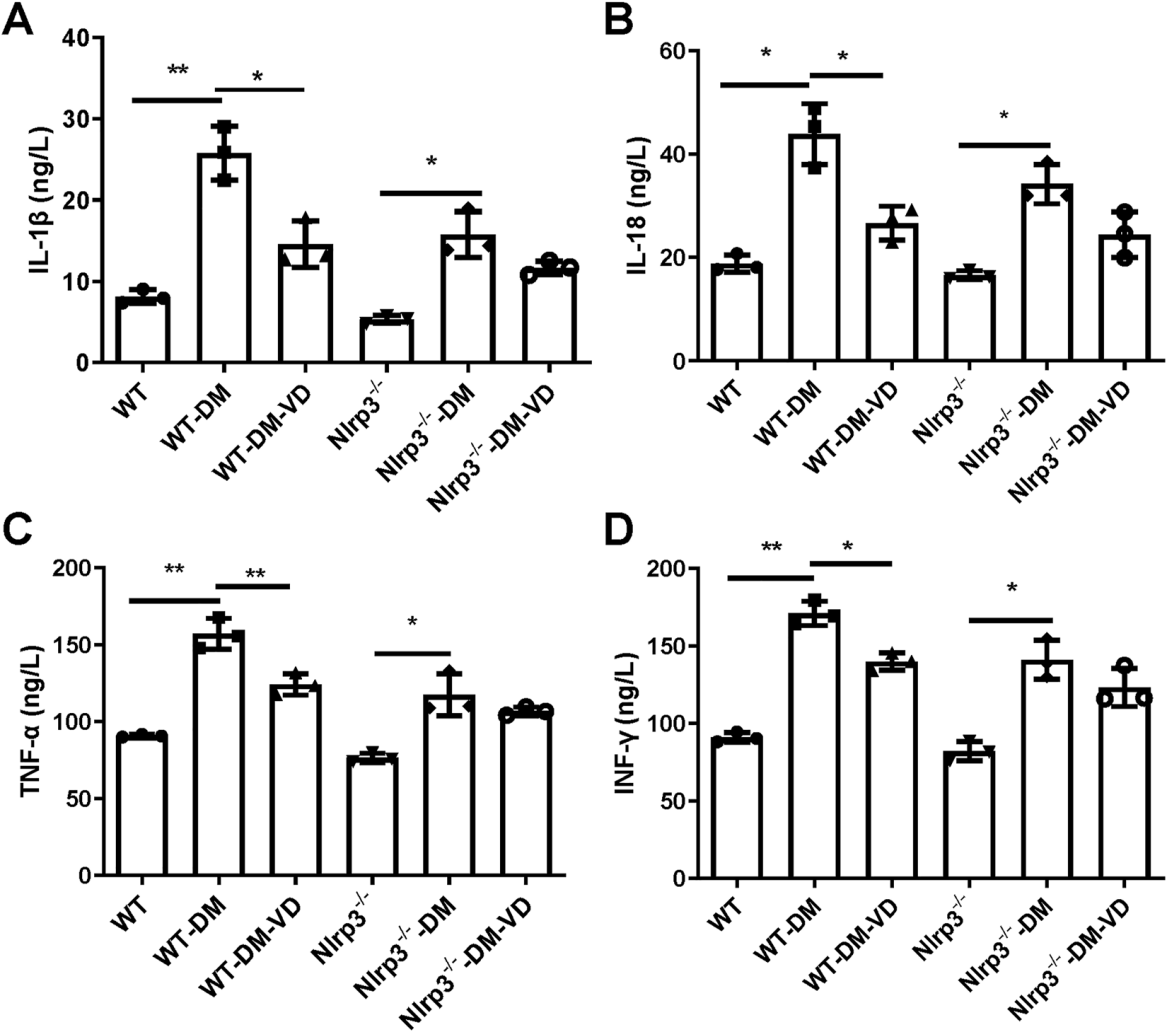
Levels of pro-inflammatory cytokines in different groups. Pro-inflammatory cytokines in the serum were detected with ELISA. (A) IL-1β. (B) IL-18. (C) TNF-α. (D) INF-γ. Note: *P < 0.05, **P < 0.01.

Serum levels of the pro-inflammatory cytokines IL-1β, IL-18, TNF-α, and IFN-γ were significantly elevated in both WT-DM and Nlrp3^−/−^-DM mice compared to their non-diabetic controls (P < 0.05; Figure 2A–D). VD supplementation significantly reduced the levels of all four cytokines in WT-DM-VD mice (P < 0.05 vs. WT-DM). In contrast, although a decreasing trend was observed, VD treatment did not lead to a statistically significant reduction in any of these cytokines in Nlrp3^−/−^-DM-VD mice compared to the Nlrp3^−/−^-DM group (P > 0.05).

### 3.3 Effects of VD on ROS levels

**Figure 3:**
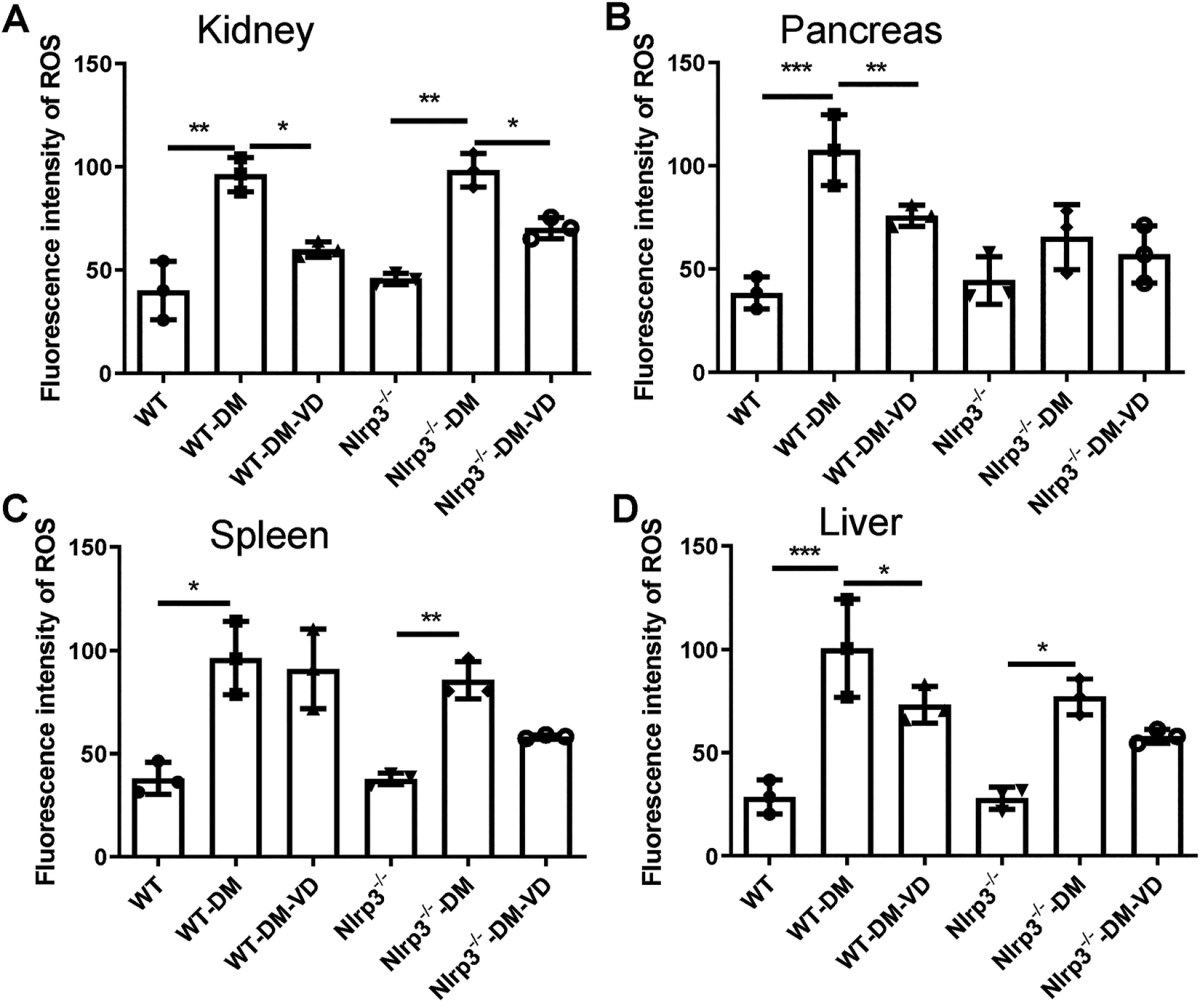
Analysis of ROS in different groups. The fluorescence intensity of ROS in the kidney (A), pancreas (B), spleen (C), and liver (D) is shown. Note: *P < 0.05, **P < 0.01, ***P < 0.001.

ROS levels were significantly increased in the kidney, pancreas, spleen, and liver tissues of WT-DM mice compared to WT controls (P < 0.05; Figure 3A–D). VD treatment significantly reduced ROS levels in the pancreas, kidney, and liver of WT-DM-VD mice (P < 0.05). In Nlrp3^−/−^-DM mice, ROS levels were also elevated in the kidney, liver, and spleen. However, VD treatment significantly lowered ROS only in the kidneys of Nlrp3^−/−^-DM-VD mice (P < 0.05 vs. Nlrp3^−/−^-DM), with no significant effect in the spleen or liver.

### 3.4 Integrated morphological analysis of pancreatic and kidney tissues

**Figure 4:**
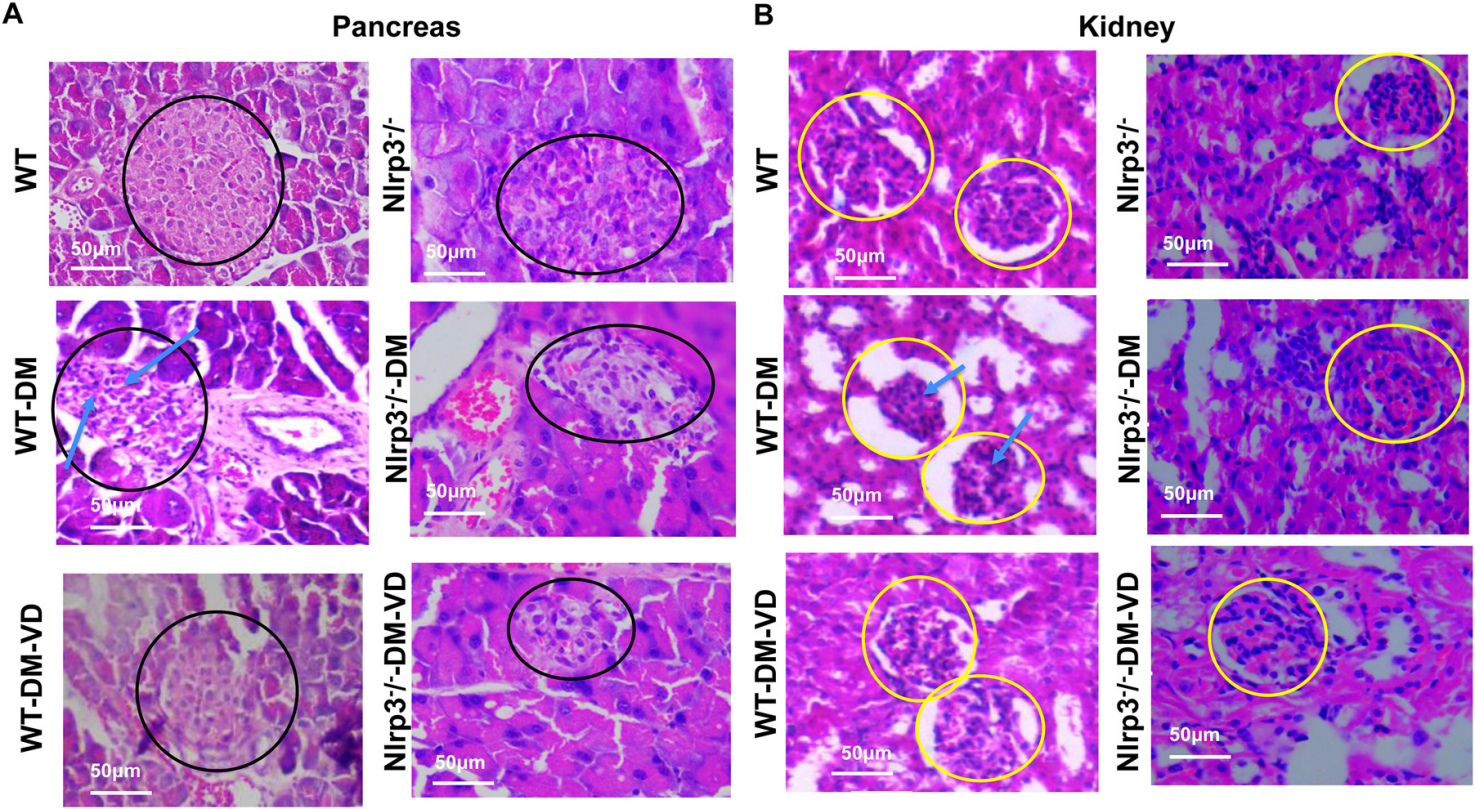
Histopathological analysis of pancreatic and kidney tissues. Representative H&E-stained sections of the pancreas (A) and kidney (B) from the indicated experimental groups. Scale bar: 50 μm. Annotations: Black circles outline pancreatic islets. Yellow circles outline kidney glomeruli. Blue arrows indicate areas of inflammatory cell infiltration in the pancreas and the kidney.

**Figure 5:**
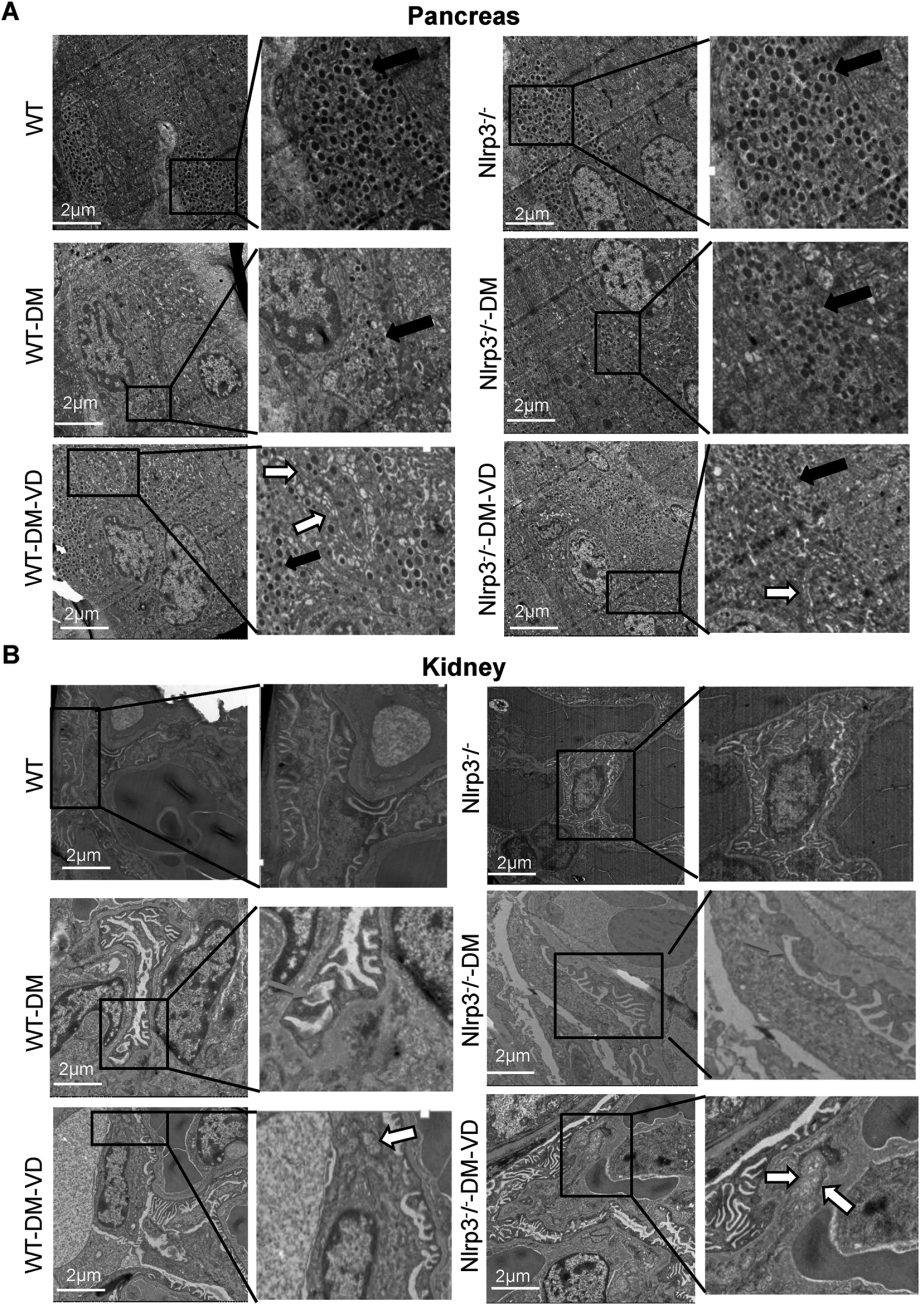
Ultrastructural analysis of pancreatic islet cells and kidney glomeruli. Transmission electron micrographs of pancreatic (A) and kidney (B) tissues from the indicated groups. Scale bar: 2 μm. Annotations: Solid black arrows point to insulin secretory granules. White arrows indicate double-membrane vesicles. Green arrows indicate effaced podocyte foot processes.

To comprehensively assess tissue and cellular integrity, we performed histopathological and ultrastructural analyses of the pancreas and kidneys. For the pancreas, histopathological examination revealed prominent, well-defined islets in control mice (Figure 4A, black circles). Islets in WT-DM mice were distorted with evident inflammatory infiltration (Figure 4A, blue arrows), while those in Nlrp3^−/−^-DM mice appeared shrunken. VD treatment preserved islet morphology in WT-DM-VD mice but showed a limited effect in Nlrp3^−/−^-DM-VD mice. At the ultrastructural level, control islet cells contained abundant electron-dense insulin secretory granules (Figure 5A, solid arrows). These granules were drastically reduced in WT-DM mice, which also exhibited swollen mitochondria with disrupted cristae. VD treatment in WT-DM-VD mice restored the number of secretory granules and led to the appearance of double-membrane vesicles (Figure 5A, white arrows). Similar autophagic structures were observed in Nlrp3^−/−^-DM-VD mice.

For the kidney, H&E staining showed normal glomeruli in controls (Figure 4B, yellow circles). WT-DM kidneys displayed glomerular hypertrophy, hypercellularity, and inflammation (Figure 4B, blue arrows), pathologies that were ameliorated by VD treatment. Nlrp3^−/−^-DM mice exhibited milder changes, with no obvious additional benefit from VD. Ultrastructurally, control podocyte foot processes were intact (Figure 5B). WT-DM mice showed extensive foot process effacement (Figure 5B, green arrows) and endothelial swelling. These damages were less severe in WT-DM-VD mice, where double-membrane vesicles were also present. Nlrp3^−/−^-DM mice showed some foot process fusion, with minimal improvement after VD treatment.

### 3.5 Expression of autophagy- and inflammation-related proteins

The expression of key proteins regulating autophagy and inflammation was analyzed in kidney and pancreatic tissues.

#### 3.5.1 Kidney

**Figure 6:**
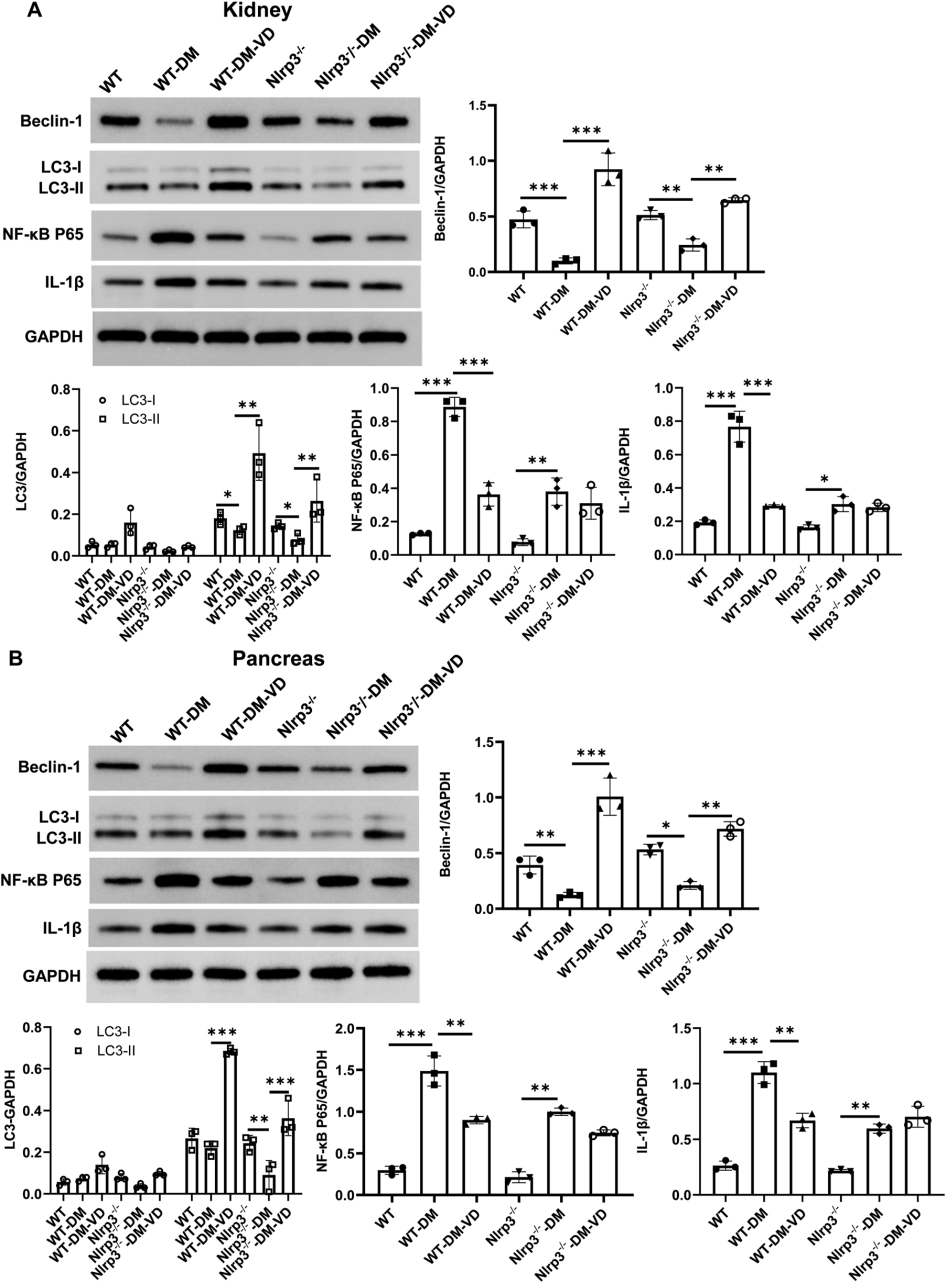
Expression of autophagy- and inflammation-related proteins in the kidney and pancreatic tissues of mice in different groups. Protein expressions of Beclin-1, LC3-I/II, NF-кB P65, and IL-1β were analyzed using Western blot. (A) Representative and quantitative Western blot results of proteins in the kidney tissues. (B) Representative and quantitative Western blot results of proteins in the pancreatic tissues. Note: *P < 0.05, **P < 0.01, ***P < 0.001.

Compared to WT controls, the expression of the autophagy-related proteins Beclin-1 and LC3-II was significantly reduced in WT-DM mice, while the inflammation-related proteins IL-1β and NF-κB p65 were significantly increased (P < 0.05; Figure 6A). VD treatment in WT-DM-VD mice reversed these changes, significantly upregulating Beclin-1 and LC3-II and downregulating IL-1β and NF-κB p65 (P < 0.05). In Nlrp3^−/−^-DM mice, LC3-II and Beclin-1 levels were also lower than in Nlrp3^−/−^ controls, and IL-1β and NF-κB p65 were elevated. VD treatment increased Beclin-1 and LC3-II expression in Nlrp3^−/−^-DM-VD mice (P < 0.05) but did not significantly reduce IL-1β or NF-κB p65 levels (P > 0.05).

#### 3.5.2 Pancreas

A similar expression pattern was observed in pancreatic tissues (Figure 6B). WT-DM mice showed decreased Beclin-1 and LC3-II and increased IL-1β and NF-κB p65 compared to WT controls (P < 0.05). VD treatment normalized these alterations in WT-DM-VD mice (P < 0.05). In Nlrp3^−/−^-DM-VD mice, VD increased autophagy markers but failed to significantly suppress the elevated inflammatory markers.

These results demonstrate that VD restores autophagy and suppresses inflammation in the pancreas and kidneys of WT diabetic mice, effects that are largely attenuated in the absence of NLRP3.

## 4 Discussion

T2DM is a chronic metabolic disorder characterized primarily by inflammation and IR [30], often accompanied by mitochondrial dysfunction and increased ROS [31]. While the beneficial effects of VD and the involvement of oxidative stress, NLRP3 inflammasome, autophagy, and NF-κB in T2DM pathogenesis have been individually recognized, the causal relationships and hierarchical dependencies among these pathways in mediating VD’s therapeutic action remain unclear. Our study directly addressed this gap. By employing NLRP3-knockout mice in conjunction with VD intervention, we genetically demonstrated that the NLRP3 inflammasome was a critical and required mediator for VD to exert its key anti-diabetic effects, which included improving glucose metabolism, reducing inflammation, and enhancing autophagic activity. This work establishes a causal link from VD action to metabolic improvement via the NLRP3 pathway, moving the field from correlation to mechanism.

Our knockout studies genetically demonstrate the crucial role of the NLRP3 inflammasome in mediating the therapeutic effects of VD. This conclusion is directly supported by our key finding that the significant improvements in glycemic control and tissue integrity observed in VD-treated WT diabetic mice were markedly attenuated in Nlrp3^−/−^ mice. These data confirm NLRP3 as a vital molecular target for VD’s action, aligning with clinical data showing that VD3 supplementation reduces NLRP3 gene expression in T2DM patients [23] and with established models where NLRP3 activation is a key trigger of obesity-induced inflammation and IR [32], [33], [34]. Notably, the partial retention of certain protective effects – specifically, the upregulation of Beclin-1 and the appearance of autophagosomal structures in Nlrp3^−/−^-DM-VD mice – suggests the involvement of ancillary, NLRP3-independent pathways. This is plausible, as direct genomic actions of VD via the vitamin D receptor (VDR) are known to modulate autophagy; for instance, VD has been shown to induce autophagy in pancreatic β-cells and enhance insulin secretion [35]. The potential crosstalk between VDR-mediated autophagy and inflammasome pathways likely contributes to the overall improvement in glucose homeostasis, a dynamic interplay that is central to metabolic regulation [36]. Furthermore, the metabolic benefits observed here corroborate earlier clinical findings that VD3 supplementation improves insulin sensitivity [37], underscoring the translational relevance of our mechanistic insights within the broader context of diabetes pathophysiology.

Mechanistically, VD initiates its protective effects by mitigating oxidative stress, as evidenced by the significant reduction in tissue ROS levels in WT-DM mice. Since ROS are potent activators of the NLRP3 inflammasome, this antioxidant action directly leads to the inhibition of NLRP3 activation and the subsequent reduction in caspase-1-mediated IL-1β maturation. The downregulation of this key pro-inflammatory cytokine, a direct interferer with insulin signaling, disrupts a major driver of IR. Our observation of concurrently decreased serum levels of IL-18, TNF-α, and IFN-γfurther supports a broad suppression of NLRP3-dependent and NF-κB-driven inflammatory pathways, collectively contributing to systemic inflammatory downregulation.

Concurrently, VD exerts a profound effect on autophagy, a process essential for cellular homeostasis and the clearance of damaged organelles like mitochondria – a primary source of ROS. Our Western blot analysis revealed that VD treatment significantly upregulated the key autophagy markers LC3-II and Beclin-1, a critical initiator of autophagosome formation whose upregulation is a recognized response to VD [35], in the tissues of WT-DM mice. The increase in these markers, together with the observation of autophagosomal structures via electron microscopy, strongly suggests enhanced autophagic activity. This enhanced activity likely contributes to the therapeutic effects via a dual-purpose mechanism. First, it facilitates the efficient removal of dysfunctional mitochondria (mitophagy), thereby further limiting a major source of ROS production and breaking the cycle of oxidative stress and inflammation [12], 16]. Second, enhanced autophagy promotes β-cell survival and function by alleviating cellular stress [13], 17]. We note that our study did not assess the autophagy substrate p62/SQSTM1; therefore, while the data indicate increased autophagic activity, they do not definitively distinguish between increased autophagosome formation and impaired lysosomal degradation. The impaired metabolic response in Nlrp3^−/−^ mice suggests intricate crosstalk, where NLRP3 inhibition might be a prerequisite for VD to fully enhance autophagic activity, or that these pathways operate in a synergistic feedback loop.

Furthermore, VD’s anti-inflammatory actions extend to the suppression of the NF-κB pathway, as indicated by the downregulation of its key component p65 and the downstream cytokine TNF-α in our study. Since NF-κB is both a master inflammatory regulator and a potent inducer of NLRP3 expression [38], its inhibition by VD likely disrupts a positive feedback loop that amplifies inflammation. This coordinated suppression of both the NLRP3 and NF-κB pathways underscores VD’s multi-faceted anti-inflammatory mechanism in T2DM.

## 5 Limitations and future perspectives

This study has several limitations that should be considered when interpreting the results and that define clear directions for future research. First, the activity of the VDR, the primary mediator of VD’s genomic effects, was not examined. Future work should quantify VDR expression or employ genetic models to link VD signaling to the observed modulation of the ROS-NLRP3-autophagy axis. Second, our findings were based on a single VD dose; dose-response studies are needed to define an optimal regimen. Third, the histopathological and ultrastructural analyses were qualitative. Although these assessments were conducted in a blinded manner using standard criteria to minimize observer bias, the incorporation of quantitative morphometry (e.g., β-cell area, glomerular diameter, infiltration index), immunohistochemistry for key targets (e.g., Beclin-1, NLRP3), and more specific autophagosome detection methods would strengthen the spatial and cellular resolution of our findings. Finally, investigating other inflammasomes (e.g., NLRC4, AIM2) in the knockout model would further confirm the specificity of the NLRP3 pathway. Additionally, the interpretation of autophagy data would be strengthened in future studies by including markers of autophagic flux such as p62/SQSTM1. Addressing these points will facilitate the translation of these mechanistic insights.

## 6 Conclusions

In conclusion, our work identifies the NLRP3 inflammasome as a critical mediator of VD’s anti-diabetic effects. We propose a cohesive model wherein VD ameliorates T2DM by: 1) reducing tissue oxidative stress, 2) inhibiting the NLRP3-IL-1β axis, 3) promoting autophagic activity to mitigate cellular damage, and 4) suppressing the NF-κB pathway. These interconnected actions collectively improve glucose homeostasis, reduce inflammation, and preserve tissue integrity in an NLRP3-dependent manner. This study provides a compelling mechanistic rationale for VD supplementation in T2DM management.
